# Nomogram based on clinical and preoperative CT features for predicting the early recurrence of combined hepatocellular-cholangiocarcinoma: a multicenter study

**DOI:** 10.1007/s11547-023-01726-2

**Published:** 2023-09-25

**Authors:** Chao Zheng, Xin-tao Gu, Xiao-li Huang, Yu-chen Wei, Lu Chen, Ning-bin Luo, Hua-shan Lin, Liao Jin-yuan

**Affiliations:** 1 Department of Radiology, The First Affiliated Hospital of Guangxi Medical University, No. 6 Shuangyong Road, Nanning, 530021 Guangxi People’s Republic of China; 2 Department of Radiology, Guangxi Medical University Affiliated Cancer Hospital, No. 71 Hedi Road, Nanning, 530021 Guangxi People’s Republic of China; 3 Department of Pharmaceutical Diagnosis, GE Healthcare, Changsha, 410005 People’s Republic of China; 4 Department of Radiology, The People’s Hospital of Guangxi Zhuang Autonomous Region, Guangxi Academy of Medical Sciences, No. 6 Taoyuan Road, Nanning, 530021 Guangxi People’s Republic of China

**Keywords:** Combined hepatocellular-cholangiocarcinoma, CT nomogram, Early recurrence, Prognosis

## Abstract

**Purpose:**

To establish and validate a multiparameter prediction model for early recurrence after radical resection in patients diagnosed with combined hepatocellular-cholangiocarcinoma (cHCC-CC).

**Materials and methods:**

This study reviewed the clinical characteristics and preoperative CT images of 143 cHCC-CC patients who underwent radical resection from three institutions. A total of 110 patients from institution 1 were randomly divided into training set (*n* = 78) and testing set (*n* = 32) in the ratio of 7–3. Univariate and multivariate logistic regression analysis were used to construct a nomogram prediction model in the training set, which was internally and externally validated in the testing set and the validation set (*n* = 33) from institutions 2 and 3. The area under the curve (AUC) of receiver operating characteristics (ROC), decision curve analysis (DCA), and calibration analysis were used to evaluate the model’s performance.

**Results:**

The combined model demonstrated superior predictive performance compared to the clinical model, the CT model, the pathological model and the clinic-CT model in predicting the early postoperative recurrence. The nomogram based on the combined model included AST, ALP, tumor size, tumor margin, arterial phase peritumoral enhancement, and MVI (Microvascular invasion). The model had AUCs of 0.89 (95% CI 0.81–0.96), 0.85 (95% CI 0.70–0.99), and 0.86 (95% CI 0.72–1.00) in the training, testing, and validation sets, respectively, indicating high predictive power. DCA showed that the combined model had good clinical value and correction effect.

**Conclusion:**

A nomogram incorporating clinical characteristics and preoperative CT features can be utilized to effectively predict the early postoperative recurrence in patients with cHCC-CC.

## Introduction

Combined hepatocellular-cholangiocarcinoma (cHCC-CC) is a rare primary liver carcinoma primarily consisting of hepatocellular carcinoma (HCC) and intrahepatic cholangiocarcinoma (ICC) [1]. The incidence of cHCC-CC varies from 0.4 to 14.2% across different regions [2–4]. Surgical resection is currently the radical treatment for cHCC-CC, but due to its high invasiveness and poor prognosis, the early postoperative recurrence rates are as high as 57–75% [5]. Since the incidence of cHCC-CC is low and the clinical-imaging features are similar to HCC, there is no staging system for cHCC-CC currently, the prognostic system for cHCC-CC mainly relies on the staging systems of HCC. However, these staging systems were developed for HCC, so they still have many shortcomings for clinical management decisions and prognosis of cHCC-CC [6–8].

In the past, numerous models for predicting postoperative recurrence of HCC have been developed based on clinical, pathological, and radiomics characteristics [9–11]. However, due to the rarity of cHCC-CC, only a few recurrence prediction models have been specifically developed for this cancer. Previous studies have identified various independent predictors of postoperative recurrence of cHCC-CC, including CA19-9 > 37 U/ml, tumor number, tumor size > 5 cm, microvascular invasion (MVI), peritumoral enhancement in the arterial phase, delayed enhancement, satellite lesions, lymph node metastasis, Mid-kine, and poorly differentiated tumors [12–19]. Although these findings are informative, most of them are from single-center studies, requiring further verifications. Furthermore, early recurrence, which mainly occurs within the initial two years after surgery and has a high recurrence rate of 57–75%, requires particular attention in cHCC-CC management [15–18, 20, 21]. A recent study developed a model for predicting very early recurrence (i.e., recurrence within 6 months after surgery) of cHCC-CC, However, it lacks preoperative imaging features, which is an important prognostic information [22].

Given the rarity and poor prognosis of cHCC-CC, identifying the risk factors that influence the prognosis of cHCC-CC and predict its recurrence is crucial. This study aims to analyze the relationship between clinical, computed tomography (CT), and pathological features of cHCC-CC and early recurrence by collecting multicenter data from three institutions. In addition, the study aims to establish and validate a scoring model for early recurrence after cHCC-CC surgery to accurately predict individualized early recurrence and guide clinical decision-making.

## Materials and methods

### Patients

**Fig. 1:**
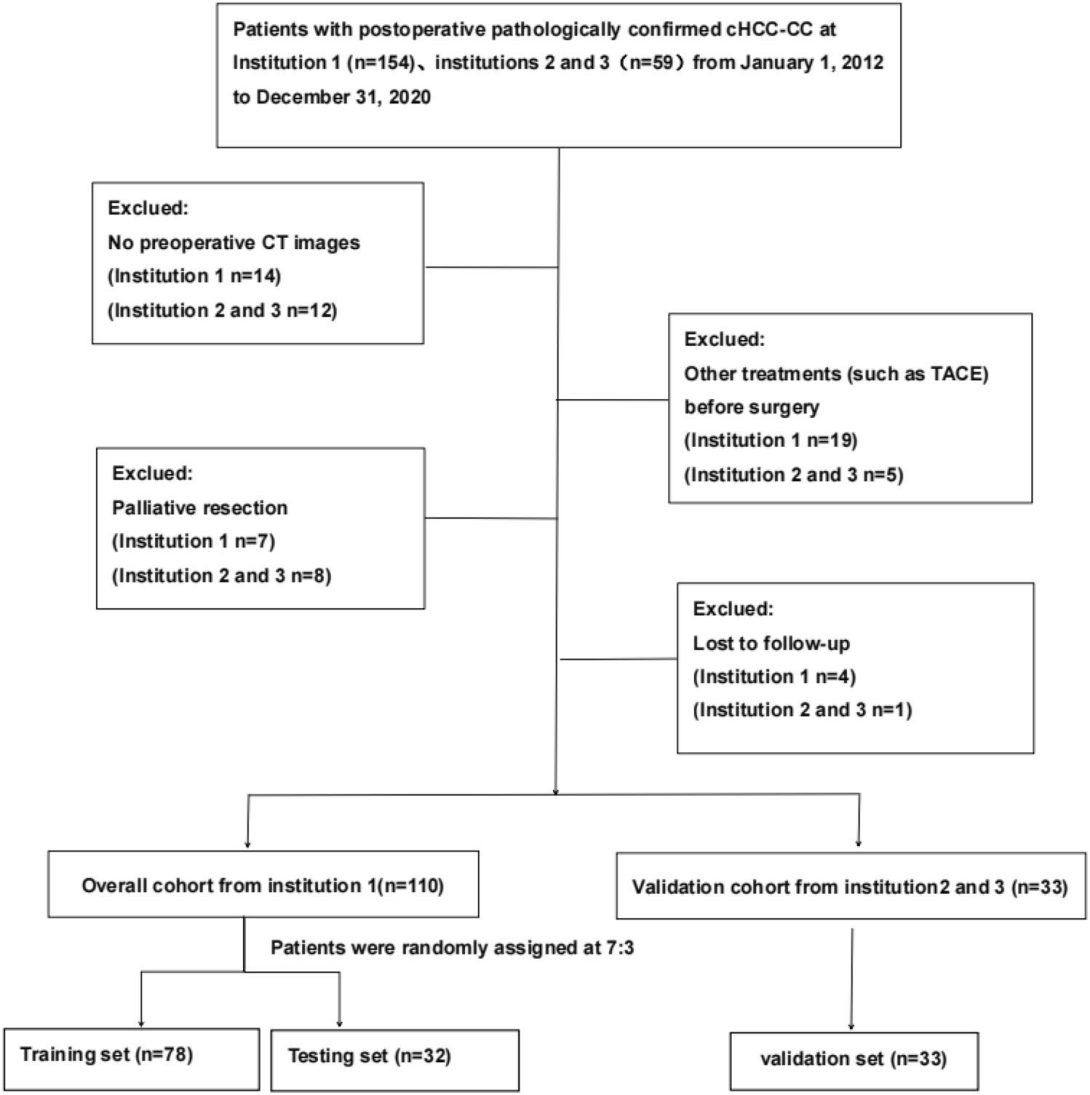
The flowchart of the inclusion process. Numbers in parentheses are numbers of patients. *Note* cHCC-CC, combined hepatocellular-cholangiocarcinoma; CT, computed tomography

This is a retrospective study in accordance with the Declaration of Helsinki and was approved by the Institutional Review Board (Approve Number: 2023-E423-01) of our hospital that waived the requirement of an informed consent. From January 1, 2012, to December 31, 2020, a retrospective analysis was conducted on 213 cHCC-CC patients confirmed by surgical pathology in three institutions (The 1st affiliated hospital of Guangxi medical University, People’s Hospital of Guangxi Zhuang Autonomous Region and Guangxi Medical University Affiliated Cancer Hospital). The inclusion criteria for patient selection were as follows: (1) presence of a single intrahepatic lesion; (2) confirmation of cHCC-CC through surgical pathology; (3) negative marginal state of resection after radical operation as confirmed under the microscope (R0); (4) high-quality enhanced CT images were obtained within 1 month; and (5) regular follow-up imaging examinations (such as ultrasound, CT, and MRI.) and laboratory examinations within 1 year after operation. The exclusion criteria included: (1) previous treatment for cHCC-CC before operation; (2) adjuvant therapy and extra-hepatic metastasis after operation; (3) loss to follow-up or died of other diseases during the follow-up; and (4) incomplete CT imaging data. (Fig. 1).

After applying inclusion and exclusion criteria, a total of 143 patients were included in the study. Therein, 110 patients from institution 1 (The 1st affiliated hospital of Guangxi medical University) were randomly divided into training set (*n* = 78) and testing set (*n* = 32) in the ratio of 7–3. Additionally, a validation cohort was formed (n = 33) from institutions 2 and 3 (People’s Hospital of Guangxi Zhuang Autonomous Region and Guangxi Medical University Affiliated Cancer Hospital).

## Follow-up

Early recurrence was defined as the occurrence of a new tumor within a year after radical resection of cHCC-CC. The starting point was the day of the operation, while the endpoint was the detection of intra- and extra-hepatic recurrence within a year post-surgery. All cHCC-CC patients were regularly monitored for 12 months after operation. Within the first six months post-surgery, monthly re-examinations were conducted. Afterward, re-examinations were scheduled every three to 6 months. The routine follow-up protocols include imaging examinations such as CT and MRI and US, and tumor markers such as serum AFP, abnormal prothrombin, AFP-L3, and other tumor markers. If new liver lesions were detected through imaging examinations like ultrasound, CT, MRI or confirmed through puncture or surgical pathological examination, the follow-up ended. The deadline of follow-up was January 31, 2023.

## Clinical, pathological data

The clinical data consist of several factors such as age, gender, hepatitis B surface antigen (HBsAg), alpha-fetoprotein (AFP), carcinoembryonic antigen (CEA), carbohydrate antigen 19-9 (CA19-9), glutamyl transpeptidase (GGT), Alanine aminotransferase (ALT), aspartate aminotransferase (AST), alkaline phosphatase (ALP), albumin (ALB), total protein (TP), prealbumin (PA), total bilirubin (TBIL), direct bilirubin (DBIL), and indirect bilirubin (IBIL). The pathological data were also collected, including MVI, hepatocyte, glypican-3, CD34, CK19, and Ki67.

## CT examination

The CT scanners were the GE64 VCT and Siemens Dual Source CT in Institution 1; Siemens Sensation 64CT and Philips iCT256 in Institution 2; and Siemens Sensation 64CT and GE Discovery CT750 in Institution 3. After a plain CT scan, an enhanced scan was performed. A high-pressure syringe was used to inject 1.5 ml/kg of non-ionic contrast agent (300 mgI/ml) at a rate of 3 ml/s through the antecubital vein. The scanning process included the arterial phase (25–30 s), venous phase (55–60 s), and equilibrium period (120 s). The slice thickness was 2 mm, the tube voltage was 120 kV, the tube current was 280 mA, and the rotation speed was 0.5 s per rotation.

## Image analysis

The CT images were review independently by two radiologists with ten years of experience, and any discrepancies were resolved through consultation between the two or with a senior doctor who has twenty years of work experience. CT features include: (1) the measurement of the tumor size in the portal phase. (2) Assessing whether the tumor margin was smooth (smooth border) or non-smooth (lobulated or irregularly protruding) as it intrudes into the surrounding normal liver parenchyma. (3) Evaluating whether the tumor has a enhancing capsule, and whether the enhancing capsule was complete (none, complete, or incomplete). The enhancing capsule was defined as uniform and smooth enhancement around the tumor and was evaluated at its evident stage (in the venous phase or equilibrium phase). A complete capsule was defined as continuous coverage of more than 80% of the tumor. (4) The peritumoral enhancement in arterial phase was also determined (none or yes), which was defined as the presence of obvious enhancement in the peritumoral liver parenchyma in the arterial phase and isodensity in the portal venous phase and equilibrium phase. (5) Intratumoral necrosis was identified (none, < 25%, 25%-50%, 51%-75%, > 75%), defined as a low-density area within the tumor without enhancement on enhanced scan. (6) The presence of peritumoral satellite lesions (absent or present) was also recorded, which were defined as cancer lesions that were less than 2 cm from the tumor margin and less than 2 cm in diameter. (7) The dilation of bile duct (none or yes) was examined, defined as low-density conduits accompanying the blood vessels in portal venous phase. (8) Large vein invasion (none or yes) was evaluated, defined as portal vein or hepatic vein filling defect in the venous phase of enhanced scanning. (9) Hepatic porta and para-aortic lymph node enlargement (absent or present) were also noted, defined as lymph node short diameter of 1 cm or more. (10) Finally, the enhancement mode (wash in and wash out, inhomogeneous enhancement, or persistence enhancement) was identified [23].

## Statistical analysis

The data were analyzed using SPSS 25.0 and R software (Version 41.0 http://www.Rproject.org). The clinical, CT, and pathological data were compared using the Chi-square and Fisher’s exact tests. Univariate and multivariate logistic regression were used to screen out the features, which were then used to construct the clinical, CT, pathological, clinical-CT, and clinical-CT-pathological combined models. The performance of the models was verified using both the testing and validation sets. The “pROC” package was used to draw the ROC curve and calculate the sensitivity and specificity of each model. The “mda” program package was used to draw the DCA curves to verify the clinical utility of the model. Nomograms and scale curves were drawn using the “rms” package according to the postoperative combined model. The correlation plot of predicted probability versus actual result was drawn using the “ModelGood” package. A statistical significance level of *P* < 0.05 was used.

## Result

### Patient characteristics

A total of 143 cHCC-CC patients were included in this study, of which 80 cases (55.9%) experienced relapse within 1 year. Among these, in the training group (*n* = 78), 42 cases (53.8%) relapsed within 1 year; in the testing group (*n* = 32), 17 cases (53.1%) experienced relapse within 1 year; and in the validation group (*n* = 33), 21 cases (63.6%) encountered relapse within 1 year. The clinical, CT, and pathological features of the cHCC-CC patients in the training, testing, and validation groups are presented in Tables 1, 2, and 3, respectively.

**Table 1:** Comparison of clinical baseline data in training set, testing set and validation set (%)

Features		Training set (*n* = 78)	Testing set (*n* = 32)	validation set (*n* = 33)	*P*
Gender	Male	58(74.36)	26(81.25)	24(72.73)	0.682
	Female	20(25.64)	6(18.75)	9(27.27)	
Age	≤ 50 years old	46(58.97)	22(68.75)	15(45.45)	0.159
	> 50 years old	32(41.03)	10(31.25)	18(54.55)	
HBsAg	Negative	9(11.54)	2(6.25)	13(39.39)	< 0.001*
	Positive	69(88.46)	30(93.75)	20(60.61)	
TBiL	≤ 20.5 umol/L	62(79.49)	26(81.25)	26(78.79)	0.967
	> 20.5 umol/L	16(20.51)	6(18.75)	7(21.21)	
DBiL	≤ 6.8 umol/L	68(87.18)	28(87.5)	27(81.82)	0.766
	> 6.8 umol/L	10(12.82)	4(12.5)	6(18.18)	
IBiL	≤ 14.3 umol/L	59(75.64)	27(84.38)	25(75.76)	0.582
	> 14.3 umol/L	19(24.36)	5(15.63)	8(24.24)	
TP	≥ 65 g/L	64(82.05)	26(81.25)	29(87.88)	0.712
	< 65 g/L	14(17.95)	6(18.75)	4(12.12)	
ALB	< 40 g/L	30(38.46)	16(50)	9(27.27)	0.170
	≥ 40 g/L	48(61.54)	16(50)	24(72.73)	
GGT	≤ 60 U/L	45(57.69)	19(59.38)	10(30.3)	0.019*
	> 60 U/L	33(42.31)	13(40.63)	23(69.7)	
AST	> 45 U/L	20(25.64)	11(34.38)	12(36.36)	0.442
	≤ 45 U/L	58(74.36)	21(65.63)	21(63.64)	
ALT	> 60 U/L	12(15.38)	7(21.88)	11(33.33)	0.104
	≤ 60 U/L	66(84.62)	25(78.13)	22(66.67)	
ALP	≤ 125 U/L	63(80.77)	25(78.13)	28(84.85)	0.781
	> 125 U/L	15(19.23)	7(21.88)	5(15.15)	
PA	< 250 mg/L	70(89.74)	28(87.5)	22(66.67)	0.008*
	≥ 250 mg/L	8(10.26)	4(12.5)	11(33.33)	
AFP	> 400 ng/ml	38(48.72)	19(59.38)	12(36.36)	0.177
	≤ 400 ng/ml	40(51.28)	13(40.63)	21(63.64)	
CA199	≤ 37 u/ml	57(73.08)	25(78.13)	27(81.82)	0.589
	> 37 u/ml	21(26.92)	7(21.88)	6(18.18)	
CEA	≤ 5 ng/ml	69(88.46)	30(93.75)	28(84.85)	0.528
	> 5 ng/ml	9(11.54)	2(6.25)	5(15.15)	

*represents *P* < 0.05

**Table 2:** Comparison of baseline data of CT features in training set, testing set and verification set (%)

Features		Training set (*n* = 78)	Testing set (*n* = 32)	Validation set (*n* = 33)	*P*
Tumor size	> 5 cm	39(50)	16(50)	21(63.64)	0.388
	≤ 5 cm	39(50)	16(50)	12(36.36)	
Peritumoral satellite lesions	No	66(84.62)	26(81.25)	24(72.73)	0.343
	Yes	12(15.38)	6(18.75)	9(27.27)	
Tumor margin	Unsmooth	62(79.49)	24(75)	31(93.94)	0.103
	Smooth	16(20.51)	8(25)	2(6.06)	
Tumor capsule	None	49(62.82)	24(75)	29(87.88)	0.086
	Smooth	7(8.97)	2(6.25)	0(0)	
	Unsmooth	22(28.21)	6(18.75)	4(12.12)	
Intratumoral necrosis	None	20(25.64)	11(34.38)	5(15.15)	0.305
	< 25%	30(38.46)	11(34.38)	12(36.36)	
	25–50%	21(26.92)	6(18.75)	9(27.27)	
	51–75%	2(2.56)	3(9.38)	5(15.15)	
	> 75%	5(6.41)	1(3.13)	2(6.06)	
Bile duct dilatation	No	67(85.9)	29(90.63)	22(66.67)	0.020*
	Yes	11(14.1)	3(9.38)	11(33.33)	
Enhancement mode	Wash in and wash out	21(26.92)	8(25)	9(27.27)	0.799
	Inhomogeneous enhancement	51(65.38)	19(59.38)	21(63.64)	
	Persistent enhancement	6(7.69)	5(15.63)	3(9.09)	
Peritumoral enhancement in arterial phase	Yes	28(35.9)	11(34.38)	14(42.42)	0.759
	No	50(64.1)	21(65.63)	19(57.58)	
Large vein invasion	No	63(80.77)	27(84.38)	19(57.58)	0.015*
	Yes	15(19.23)	5(15.63)	14(42.42)	
Lymph node enlargement	No	65(83.33)	30(93.75)	19(57.58)	0.001*
	Yes	13(16.67)	2(6.25)	14(42.42)	

*represents *P* < 0.05

**Table 3:** Comparison of baseline data of pathological features in the training set, testing set and validation set (%)

Features		Training set (*n* = 78)	Testing set (*n* = 32)	Validation set (*n* = 33)	*P*
MVI	Yes	36(46.15)	10(31.25)	18(54.55)	0.157
	No	42(53.85)	22(68.75)	15(45.45)	
Hepatocyte	Positive	57(73.08)	28(87.5)	16(48.48)	0.002*
	Negative	21(26.92)	4(12.5)	17(51.52)	
Glypican3	Negative	15(19.23)	9(28.13)	9(27.27)	0.488
	Positive	63(80.77)	23(71.88)	24(72.73)	
CD34	Positive	69(88.46)	30(93.75)	25(75.76)	0.101
	Negative	9(11.54)	2(6.25)	8(24.24)	
CK19	Positive	73(93.59)	31(96.88)	29(87.88)	0.409
	Negative	5(6.41)	1(3.13)	4(12.12)	
Ki67	> 20%	58(74.36)	20(62.5)	24(72.73)	0.449
	≤ 20%	20(25.64)	12(37.5)	9(27.27)	

*MVI* Microvascular invasion

*represents *P* < 0.05

## Model construction

A total of 33 variables encompasses clinical data, CT features, and pathological data. Through single-factor logistic regression, 10 important variables for early recurrence in cHCC-CC were identified, including AST > 45 U/L (P = 0.0013), ALT > 60 U/L (*P* = 0.0189), ALP > 125 U/L (*P* = 0.0111), tumor size > 5 cm (*P* = 0.0004), tumor margins not smooth (*P* = 0.0039), intratumoral necrosis (*P* = 0.0134), continuous enhancement of the tumor margin (*P* = 0.0168), peritumoral enhancement in the arterial phase (*P* = 0.0017), large vein invasion (*P* = 0.0325), and MVI (*P* = 0.0118). Incorporating these indicators into multivariate logistic regression analysis revealed that AST > 45 U/L, ALP > 125 U/L, and MVI were independent risk factors for early recurrence in cHCC-CC patients. Both univariate and multivariate logistic regression analyses showed that AST had the highest odds ratio for early recurrence of cHCC-CC after radical resection (Table 4).

**Table 4:** The results of univariate logistic regression analysis and multivariate logistic regression analysis of each variable in the training set

	Univariate logistic regression analysis		Multivariate logistic regression analysis	
	OR(95%CI)	*P*	OR(95%CI)	*P*
Gender	2.83(0.98–8.15)	0.054		
Age	0.77(0.31–1.90)	0.57		
HBsAg	0.29(0.06–1.52)	0.144		
TBiL	3.20(0.93–11.02)	0.065		
DBiL	1.33(0.35–5.15)	0.677		
IBiL	3.10(0.99–9.71)	0.052		
TP	1.69(0.51–5.60)	0.390		
ALB	0.97(0.39–2.41)	0.943		
GGT	2.00(0.80–5.02)	0.140		
AST	12.75(2.70–60.16)	0.001*	9.73(1.89–50.04)	0.006*
ALT	12.42(1.52–101.68)	0.019*	4.89(0.49–48.60)	0.176
ALP	7.62(1.59–36.59)	0.011*	7.52(1.43–39.54)	0.017*
PA	2.10(0.46–9.46)	0.336		
AFP	2.10(0.85–5.19)	0.110		
CA199	2.07(0.73–5.89)	0.172		
CEA	0.38(0.09–1.67)	0.201		
Number of tumors	2.48(0.78–7.89)	0.124		
Tumor size	5.80(2.18–15.45)	< 0.001*	2.95(0.97–8.98)	0.057
Peritumoral satellite lesions	3.00(0.75–12.08)	0.122		
Tumor margin	7.35(1.89–28.54)	0.004*	3.64(0.84–15.83)	0.085
Tumor capsule	1.26(0.76–2.10)	0.367		
Intratumoral necrosis	1.88(1.14–3.11)	0.013*		
Bile duct dilatation	1.60(0.43–5.98)	0.485		
Tumor enhancement	2.99(1.22–7.34)	0.017*		
Peritumoral enhancement in arterial phase	5.50(1.90–15.96)	0.002*	2.63(0.80–8.67)	0.113
Large vein invasion	4.40(1.13–17.11)	0.033*		
lymph node enlargement	143,564,018.56(0– + ∞)	0.992		
MVI	3.34(1.31–8.55)	0.012*	3.34(1.31–8.55)	0.012*
Hepatocyte	1.41(0.52–3.84)	0.504		
Glypican3	1.43(0.46–4.42)	0.536		
CD34	0.93(0.23–3.74)	0.913		
CK19	5.13(0.55–48.12)	0.153		
Ki67	2.13(0.75–5.99)	0.154		

*OR* odds ratio; *CI* confidence interval

*means *P* < 0.05

AST and ALP were used to construct the clinical model of early postoperation recurrence of cHCC-CC. The tumor size, tumor margin, and arterial phase peritumoral enhancement were used to construct the CT model of early recurrence of cHCC-CC. MVI was used as an independent predictor to construct the pathological model. AST, ALP, tumor size, tumor margin, and arterial phase peritumoral enhancement were used to construct the clinic-CT model. MVI was included to construct the clinical-CT-pathological combined models based on the clinic-CT model.

## Comparison of predictive performance of different models

**Fig. 2:**
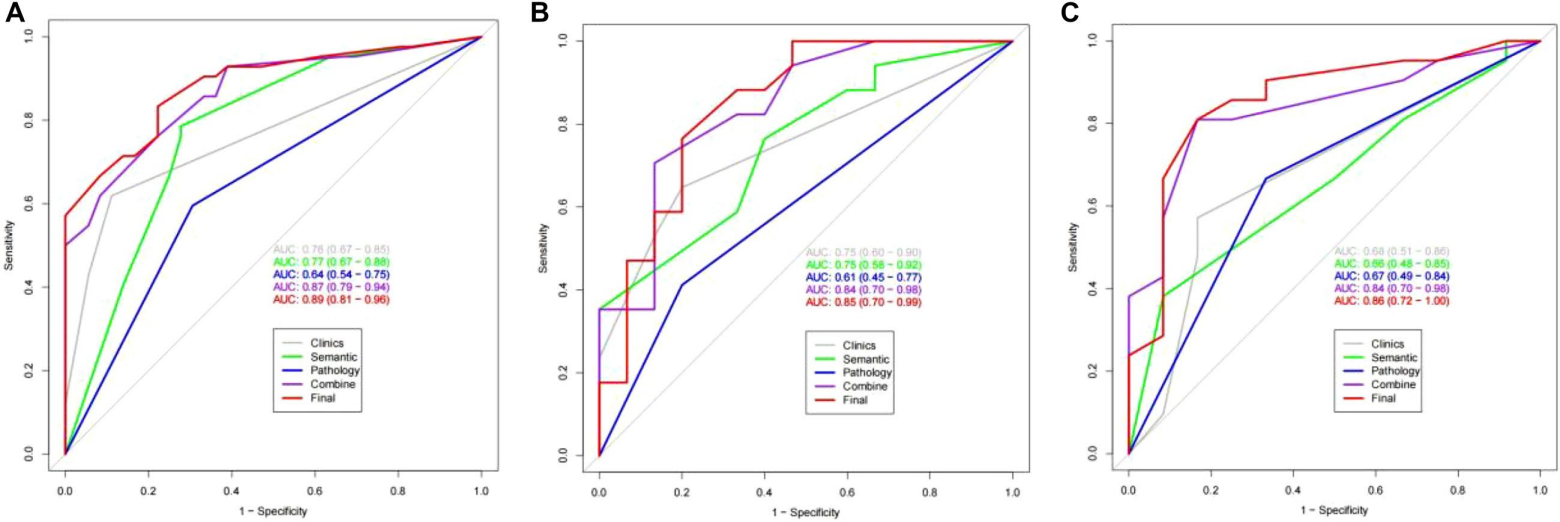
AUC curves of each model in the training set (**a**), testing set (**b**), and validation set (**c**)

**Fig. 3:**
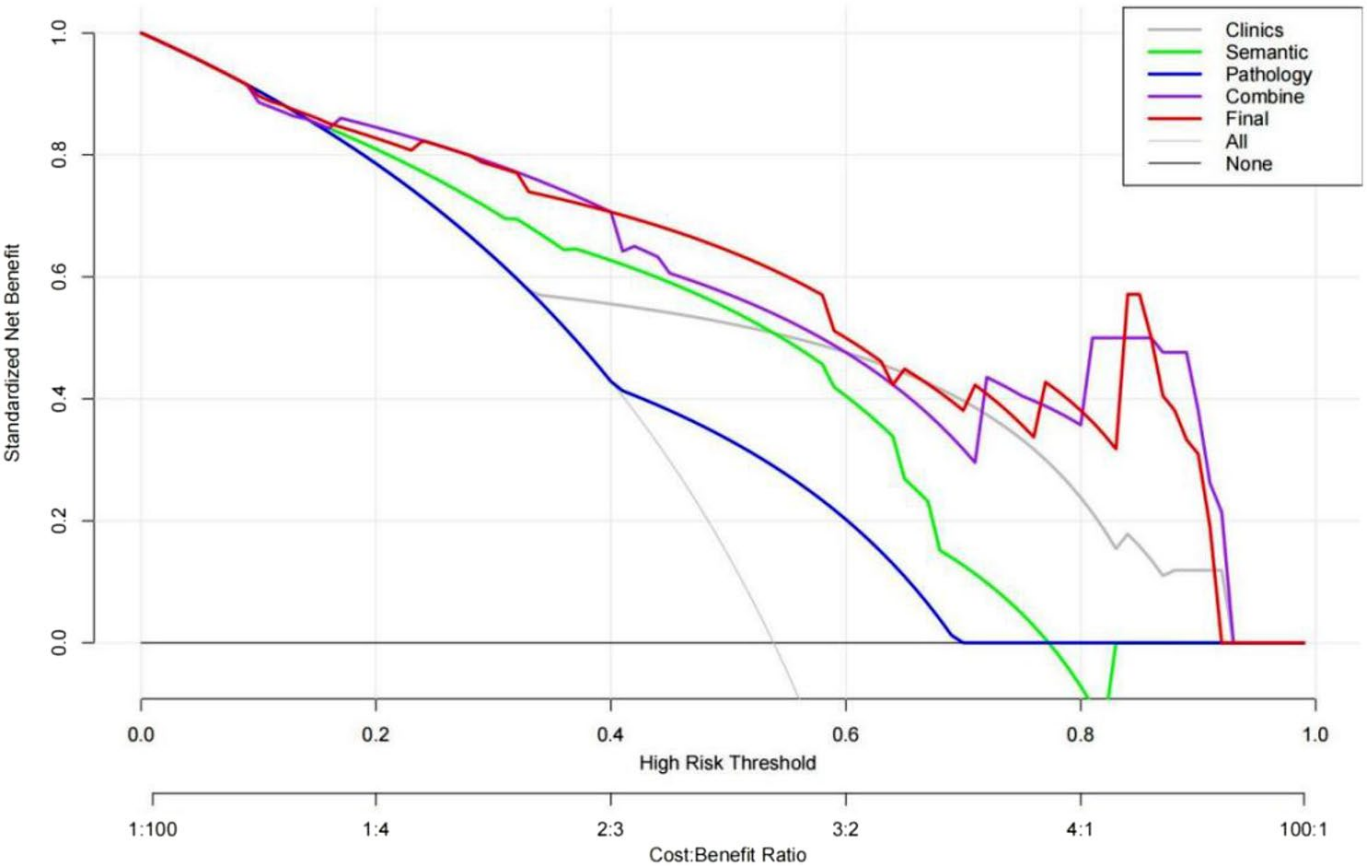
Clinical decision analysis of each model in the training set. The abscissa is the threshold probability; the ordinate is the net benefit minus the harm. The gray curve indicates that all patients received the intervention; the black horizontal line indicates that all patients did not with a net benefit of 0

The effectiveness of the five models in predicting early recurrence after cHCC-CC resection is presented in Table 5. The results indicate that the combined model outperformed other models in predicting postoperative recurrence, with a higher AUC observed in the training, testing, and validation sets (Fig. 2). Additionally, the combined model demonstrated higher net gain in clinical decision-making (Fig. 3).

**Table 5:** The effectiveness of each model in predicting the early recurrence of cHCC-CC

Model	Training set			Testing set			Validation set		
	AUC	Sensitivity (%)	Specificity (%)	AUC	Sensitivity (%)	Specificity (%)	AUC	Sensitivity (%)	Specificity (%)
Clinical model	0.76	61.90	88.89	0.75	64.71	80.00	0.68	57.14	83.33
CT model	0.77	78.57	72.22	0.75	76.47	60.00	0.66	38.09	91.67
Pathological model	0.64	59.52	69.44	0.61	41.17	80.00	0.67	66.67	66.67
clinic-CT model	0.87	92.86	61.11	0.84	70.58	86.67	0.84	80.95	83.33
Combined model	0.89	83.33	77.78	0.85	76.47	80.00	0.86	80.95	83.33

*AUC* area under the curve

## Nomogram establishment

**Fig. 4:**
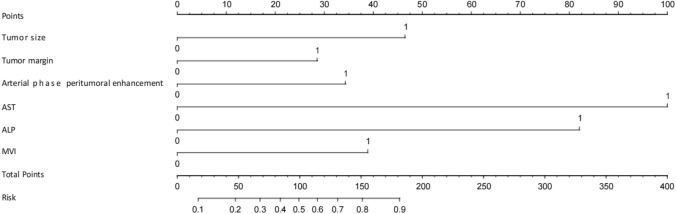
Construct a nomogram based on the combined model and add the points of each variable to obtain the total points. The total points correspond to the risk probability of early postoperative recurrence (Risk), which can be visualized Readout of the nomogram predicts a patient’s risk probability of recurrence within one year after surgery

**Fig. 5:**
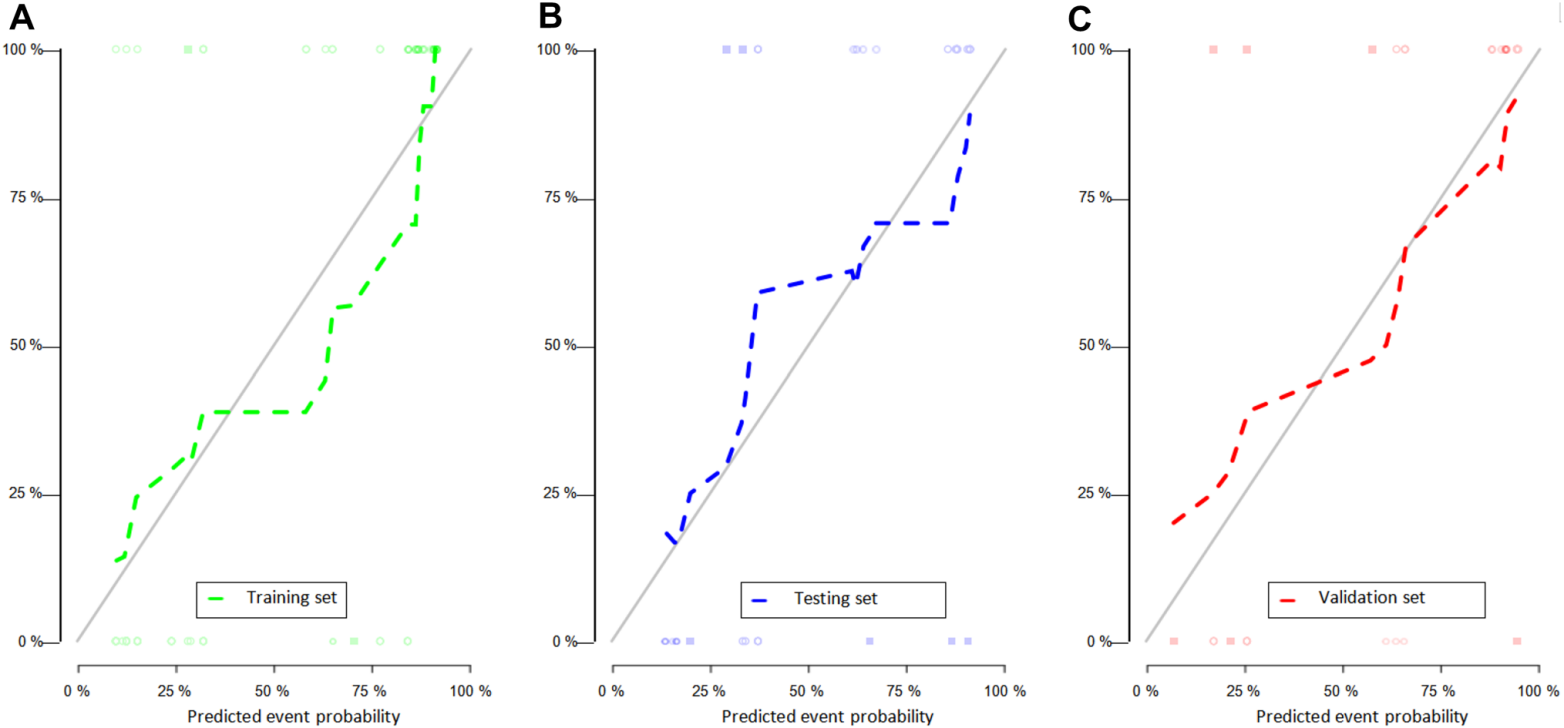
Nomogram risk prediction model calibration curve. **a** Training set; **b** testing set; **c** validation set. The solid line represents the ideal predictive performance, and the dashed line represents the predictive performance of the nomogram. The closer the dotted line is to the solid line, the better the prediction accuracy of the nomogram. Calibration analysis showed that the predicted results of early relapse in the three cohorts were agreed with the actual results

The risk prediction model using the nomogram was based on the combined model (Fig. 4). The calibration curve of the nomogram risk model demonstrated that the predicted probability was almost identical to the actual probability in the training, testing and validation sets (Fig. 5). This indicates that the nomogram model’s prediction accuracy is significantly high.

## Typical case image

**Fig. 6:**
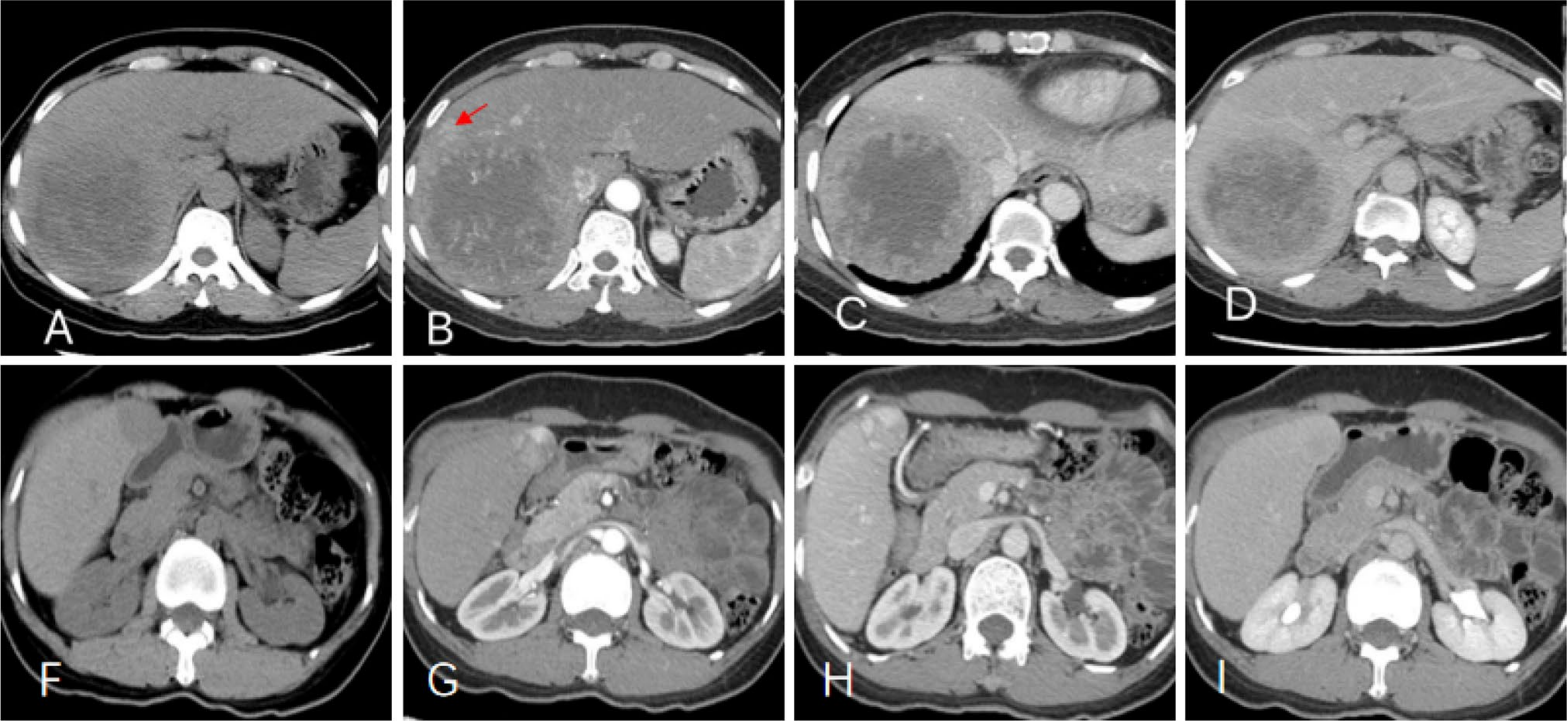
**a**–**d** a 46-year-old male cHCC-CC patient with AST 61U/L, ALP 153U/L, MVI positive. **a** axial plain scan showed the tumor size > 5 cm and inhomogeneous; **b** peritumoral enhancement (red arrow shown) in arterial phase; **c**, **d** venous phase and delay phase showed the tumor necrosis > 75%, and tumor margins was unsmooth. **f**–**i** a 48-year-old female cHCC-CC patient with AST 27U/L, ALP 33U/L, MVI negative. **f** axial plain scan showed the tumor size < 5 cm; **g** no significant peritumoral enhancement in arterial phase; **h**, **i** in the venous phase and delay phase, the degree of tumor necrosis was less than 25%, and the tumor margin was smooth

*Case 1*: A 46-year-old male cHCC-CC patient in the right lobe of the liver (Fig. 6a–d), recurrence 2 months after surgery.

*Case 2*: A 49-year-old female cHCC-CC patient in the right lobe of the liver did not recur after radical surgery in August 2014 until the end of the follow-up visit (Fig. 6f–i).

## Discussion

Although surgical resection is the main treatment for cHCC-CC, the postoperative recurrence rate remains high, as evidenced by previous studies [5, 15, 17]. More than half of cHCC-CC patients have been shown to experience early recurrence within two years [15–17, 20, 21]. Our study found that 55.9% of the 143 patients relapsed within one year, indicating a poor prognosis. To identify patients who are at high risk of recurring, it is not appropriate to use a two-year cut-off point for early recurrence. Therefore, we developed and validated a postoperative comprehensive model that involves preoperative clinical and CT features as well as postoperative pathological risk factors (the features are including AST, ALP, tumor size, tumor margin, arterial phase peritumoral enhancement, and MVI). And then a nomogram was constructed to predict early recurrence (within one year) in cHCC-CC patients who undergo radical surgery. Our study findings demonstrate the effectiveness of the nomogram in predicting early recurrence in such patients. The accuracy rates for the training set, testing set, and validation set were 0.81, 0.78, and 0.82, respectively. This tool can aid clinicians in identifying patients who are at high risk for early recurrence, enabling more robust surveillance strategies and appropriate anti-tumor strategies to be implemented.

CT is a widely used as a noninvasive imaging modality in routine clinical practice for the diagnosis, treatment planning, and monitoring of cancer. The tumor size is considered an essential factor that affects early recurrence following radical resection of cHCC-CC. This may be attributed to the fact that larger tumors exhibit more aggressive invasive biological behavior, grow at a faster rate, and are more prone to infiltrating surrounding liver tissue, penetrating the capsule, and developing intrahepatic metastasis. This finding is consistent with previous studies [13, 15].

Previous research also indicates that irregular tumor margins are significant predictors of liver cancer recurrence [24, 25], with an accuracy of 69.5% reported by Ariizumi et al. [15]. Peritumoral enhancement reflects the blood perfusion of the liver tissue surrounding the tumor. When the tumor thrombus obstructs the small branches of the portal vein around the tumor, there was a decrease in portal vein blood flow and slowed flow velocity in that area [26]. Vascular regulatory factors then regulate the vasodilatation of the branches of the hepatic artery, leading to a compensatory increase in blood flow. This results in abnormal enhancement of the liver tissue around the tumor on CT in the arterial phase. Such enhancement suggests cancer cell infiltration in the blood supply around the tumor, thus increasing the risk of cancer cell dissemination and metastasis. This finding is consistent with the author’s research.

AST and ALP have been recognized as independent risk factors for the early recurrence of cHCC-CC. Previous studies on early recurrence of HCC also highlight the independent influence of AST [27, 28] and ALP [29, 30] as contributing factors. Similarly, MVI has been established as a strong and independent predictor of early postoperative recurrence of cHCC-CC [12, 13, 15, 22], likely because it is associated with the cytokines and proteins secreted by stromal cells in the tumor microenvironment that promote angiogenesis. MVI frequently involves small branches of the portal vein or hepatic vein, which results in early blood vessel dissemination. Tumor cells in these vessels exhibit extensive vascularization, demonstrate rapid growth and have the potential to cause intrahepatic recurrence and distant metastasis [31]. These observations align with the results of the study under discussion.

Wu et al. [22] used MiVI, MaVI, and CA19-9 > 25 u/ml as independent risk factors for very early postoperative recurrence in cHCC-CC patients and constructed a nomogram model, with AUCs of 0.77 (95%CI 0.69–0.85) and 0.76 (95% CI 0.66–0.86) in the training set and validation set, respectively. Compared to that study, our study added CT features (included tumor size, tumor margin and arterial phase peritumoral enhancement) on the basis of clinical, pathological features, and the results improved the predictive power. The AUCs of the model were 0.89 (95% CI 0.81–0.96), 0.85 (95% CI 0.70–0.99) and 0.86 (95% CI 0.72–1.00) in the training, testing, and validation sets, respectively. In addition, our nomogram’s was based on the datasets from three institutions, which was internally and externally validated in testing and validation sets, and the addition of external validation improves model reproducibility and generalization compared to existing models. With this knowledge at their disposal, medical practitioners can now objectively evaluate the various parameters involved in cHCC-CC patients and choose the best treatment strategies and monitoring plans tailored to their patients, ensuring better clinical outcomes.

Our study has several limitations that need to be acknowledged. Firstly, this article is based on a retrospective study, which may lead to bias in the data. Secondly, our study focused on patients who underwent radical hepatectomy, which raises the question of whether our predictive nomogram model can be applied to patients receiving other treatments. Thirdly, near 83% patients in our group infected HBV. We all know that in Eastern countries, the majority of cHCC-CC patients are affected by HBV infection, but it occurs less frequent in western populations, so the generalizability of the conclusion maybe limited.

In conclusion, the nomogram model we constructed using six selected clinicopathologic parameters exhibits exceptional internal and external validity, demonstrating robust predictive power and serving as a valuable clinical decision-making tool with favorable net benefit and calibration.
